# Human papillomavirus clustering patterns among HIV-infected and HIV-uninfected adolescent females in South Africa

**DOI:** 10.5897/JAHR2017.0445

**Published:** 2017-10-31

**Authors:** Layne Dylla, Beau Abar, Anna-Lise Williamson, Tracy L. Meiring, Linda-Gail Bekker, David H. Adler

**Affiliations:** 1 Department of Emergency Medicine, University of Rochester, Rochester, New York, United Sates; 2 Institute of Infectious Diseases and Molecular Medicine and Division of Medical Virology, Faculty of Health Sciences, University of Cape Town, Observatory 7925, Cape Town, South Africa; 3 Institute of Infectious Diseases and Molecular Medicine, Faculty of Health Sciences, University of Cape Town, Desmond Tutu HIV Centre, Observatory 7925, Cape Town, South Africa

**Keywords:** Human papillomavirus (HPV), clustering, latent class analysis, high-risk HPV, South African adolescents

## Abstract

The global burden of disease caused by both human immunodeficiency virus (HIV) and human papillomavirus (HPV) is the greatest in the developing world, with the highest rates in sub-Saharan Africa. South African women not only have high rates of infection with HPV, but also have high rates of multiple concurrent infections with two or more HPV genotypes, and are among the world’s most vulnerable to developing invasive cervical cancer. HIV co-infection increases these risks. Understanding clustering patterns of concurrent HPV infections in this population has important implications for HPV screening and will help define vaccination strategies in the future as vaccines continue to be developed to target more HPV genotypes. Latent class analysis was used to identify four distinct patterns of HPV co-infection: individuals with at least one low risk HPV genotype, but no high-risk HPV (HR-HPV) infections; individuals with a disperse pattern of HR-HPV infections; individuals infected with members of the alpha-7 group, but not HPV-18; and individuals infected with HPV-16, but not HPV-18. In this analysis, although alpha-7 HPV infections were more prevalent among HIV-infected adolescents than their HIV-uninfected counterparts, overall clustering patterns were not different based on HIV status.

## INTRODUCTION

The high-risk human papillomavirus genotypes (HR-HPV) cause cervical cancer and are targets of vaccines preventing HPV infection and subsequent malignant transformation. While most HPV infections are transient, they are more likely to persist and progress to invasive disease when multiple concurrent infections are present and/or there is co-infection with human immunodeficiency virus (HIV) (Bello et al., 2009; Kane et al., 2012; Schlecht et al., 2001; Trottier et al., 2008). It has been shown that as compared to HIV-uninfected women, HIV-infected women are infected with a broader distribution of HPV genotypes and are more likely to be infected with multiple HPV genotypes and at increased risk of death from cervical cancer (Clifford et al., 2006; Denny et al., 2012; Massad et al., 2001; Sun et al., 1997). Additionally, infection with HR-HPV also makes women more susceptible to HIV infection (Williamson, 2015). South African women are at high risk of developing and dying from cervical cancer (Ferlay et al., 2010).

South African adolescent women have high rates of infection with multiple concurrent HPV genotypes and HIV, but only 15.4% of South African adolescents were infected with the two HPV genotypes typically covered by current vaccine strategies (HPV-16 and HPV-18) (Adler et al., 2013). Despite this relatively low HPV-16 and HPV-18 infection rates among South African adolescent women, in sub-Saharan African, adult females with invasive cervical cancer, HPV-16 and HPV-18 were among the genotypes most frequently identified in single and multiple HPV infections (Denny et al., 2012). The exact reasons for this are unclear. However, given this increased risk of cervical cancer, strides continue to be made with the development of new vaccines to target more HR-HPV genotypes, including a recently FDA-approved vaccine targeting HPV-6, 11, 16, 18, 31, 33, 45, 52, and 58. Unfortunately, developing nations like those in sub-Saharan Africa, which have a high incidence of cervical cancer, have limited capacity to implement large-scale vaccination programs due to HPV vaccine costs and issues surrounding vaccine delivery to targeted adolescent populations (Ferlay et al., 2010; Kane et al., 2012). With limited ongoing vaccination efforts in developing nations, it is imperative to understand both the distribution of HPV genotypes and their clustering patterns to ensure that vaccinations are adequately targeting regional patterns of HPV infections.

This study used a novel approach to the question, employing latent class analysis (LCA), to better understand clustering patterns of HPV. LCA is a parametric modeling procedure to determine discrete patterns of subgrouping within a population with multivariate categorical data. Compared to previous methods, such as hierarchical or k-means cluster analysis, LCA allows for statistical and conceptual comparison of multiple grouping patterns to ensure that data-clustering patterns most accurately reflect potential biological clustering, whereas previously employed methods rely largely on arbitrary stopping rules to decide the optimal number of groups, clusters or classes.

## MATERIALS AND METHODS

A cross-sectional study was conducted in which self-collected vaginal swabs for HPV DNA testing were collected from 100 sexually active HIV-infected and HIV-uninfected South African adolescent females aged 17 to 21 years between October 2012 and October 2014. Participants were recruited from the youth community center and clinic in two urban disadvantaged communities in Cape Town, South Africa. Exclusion criteria included a history of HPV vaccination and/or cervical surgery.

Informed consent was obtained from all participants aged 18 and above. Adolescent assent and parental consent were obtained for participants aged 17 years. The Research Subjects Review Board at the University of Rochester and the Human Research Ethics Committee at the University of Cape Town approved the study.

HIV status was confirmed upon enrollment. Fifty study participants were HIV-infected and 50 were HIV-uninfected. However, for the purpose of this analysis, only adolescents with at least one HPV infection at enrollment (n=64) were included. Results of HPV DNA testing of self-collected specimens were used in this analysis. Participants were instructed to twirl a Dacron swab high in the vagina for 10 s. Specimens were transported in Digene transport medium. The MagNA pure compact nucleic acid isolation kit (Roche) was used to extract the DNA. The Roche Diagnostic Linear Array HPV test was used for HPV genotyping. This research-only test identifies 37 HPV genotypes including the 13 oncogenic HR-HPV genotypes in the alpha-papillomavirus genus as designated by the International Agency for Research on Cancer: types 16, 18, 31, 33, 35, 39, 45, 51, 52, 56, 58, 59, and 68 (Straif et al., 2009).

Patterns of infections were examined using LCA. Analyses were performed using Mplus 7.11 and the best fitting model was determined by comparing the Akaike Information Criteria (AIC) and the Lo-Mendell-Rubin adjusted likelihood ratio test (LMR aLRT) across solutions with differing numbers of classes (Muthen and Muthen, 2012). LCA provides estimates of group probabilities (that is, probability/prevalence of each class modeled) and conditional probabilities (that is, the probability of a specific infection given membership in a specific class).

Based on a meta-analysis of HPV infections in African women with invasive cervical cancer in which HPV-16, HPV-18, and HPV-68 were among the top ten HR-HPV genotypes detected (Ogembo et al., 2015), these genotypes and their respective alpha-7 and alpha-9 groups were the focus of this study and used as determinants for the potential latent classes. While the alpha-papillomavirus species-7 (alpha-7) group consists of HPV genotypes: 18, 39, 45, 59, 68, 70, 85, and 97, the Roche Diagnostic Linear array does not detect HPV- 70, 85, or 97 (de Villiers, 2013). Thus, for the purposes here, the alpha-7 group was limited to HPV genotypes: 18, 39, 45, 59, and 68. The alpha-papillomavirus species-9 (alpha-9) group was defined as HPV genotypes: 16, 31, 33, 35, 52, 58, and 67(de Villiers, 2013).

## RESULTS

The demographic and behavioral characteristics of the cohort are shown in Table 1. Five separate indicators of latent classes were used: (i) HPV-16, (ii) HPV-18, (iii) HPV-68, (iv) an alpha-7 infection other than HPV-18, and (v) an alpha-9 infection other than HPV-16. A model with four patterns of infections fit the data best (Table 2), as the 4-class model demonstrated the lowest AIC value and a significant LMR aLRT (*p* = 0.05) indicating statistically better fit than the 3-class model. The first class included adolescents without a HR-HPV infection (termed Uninfected) and comprised 35% of the population. Class 2 represented the largest group of HR-HPV-infected individuals, 34% of the population, and comprised individuals with a disperse pattern of infection (termed Disperse Infections), though none of these individuals had an HPV-68 infection. These individuals had the highest conditional probabilities across classes of a non-HPV-16 alpha-9 infection (51% chance of infection), while also being the only individuals with a non-zero probability of an HPV-18 infection (37% chance of infection). Class 3 represented 18% of the population and was limited to individuals with moderate conditional probabilities of concurrent infections with HR-HPV alpha-7 genotypes (HPV- 39, 45, 59, 68) other than HPV-18 (termed HPV-68 and non-HPV-18 alpha-7). These individuals demonstrated the highest probability across classes of a non-HPV-18 alpha-7 infection (60% chance of infection). Class 4 represented 14% of the population and consisted of individuals infected with HPV-16, but not HPV-18 (termed HPV-16, not HPV-18) and had the highest probability of HPV-68 infection (57% chance of infection). There were no differences across classes in HIV status, lifetime sexual partners, frequency of condom use, use of contraceptive injections, or use of contraceptive pills (all p-values > 0.05). Analysis of HIV-infected adolescents as compared to HIV-uninfected adolescents revealed no differences in clustering patterns. However, further analysis revealed that alpha-7 infections were the only pattern of infection significantly more prevalent among HIV-infected adolescents (38% of HIV-infected adolescents as compared to 13% of HIV-uninfected adolescents, p=0.044).

## DISCUSSION

Infection with multiple HPV genotypes and/or co-infection with HIV increases the risk of progression to invasive cervical disease (Bello et al., 2009; Clifford et al., 2006; L. Denny et al., 2014; Schlecht et al., 2001; Trottier et al., 2008). Multiple studies of African women have found high rates of concurrent HPV infections (Adler et al., 2013; Menon et al., 2016; Said et al., 2009). The data presented here identified four distinct patterns of infection: (i) a group with at least one low risk HPV infection but no HR-HPV infections; (ii) a sizable group of individuals with a disperse pattern of infection and two smaller groups of individuals characterized by individuals: (iii) alpha-7 infections, with exception of HPV-18, and (iv) an HPV-16 infection, but no HPV-18 infection.

Not surprisingly given the uniqueness of this study population and the broad variations in clustering patterns in previous studies, LCA analysis revealed four new distinct patterns of infections. By using LCA rather than the non-parametric methods previously employed, this study was capable of providing statistical support for asserting that the patterns identified most accurately reflect potential biological clustering.

In a study of US women, HPV-16 was the most frequently detected genotype in both single infections and in infections with multiple HPV genotypes (Spinillo et al., 2009). It also found that HPV-16-18, HPV-51-52, HPV 31-51-56, and HPV 16-51-52 were the only patterns of HPV infections present at increased rate observed to expected ratios. Similarly, in a previous study of adult South African women, 90.4% of cervical cancer samples analyzed were co-infected with HPV-16 and HPV-16 was the most frequent genotype identified (88.5% of all HPV infections) (Lebelo et al., 2015). However, in these adult South African women, HPV-18 was only the third most frequent infection (20% of all HPV infections) and HPV-68 was the only HR-HPV genotype never isolated. In South African adolescents presented here, HPV-16 co-infections were also observed frequently (10 of 13 HPV-16 infections occurring in consort with another HR-HPV infection).

In the current study, HPV-16 infections clustered in one of two classes with other disperse HR-HPV infections or with individuals who were not co-infected with HPV-18, but had a relatively high probability of infection with HPV-68. HPV-18 only clustered in individuals with a substantial probability of a disperse pattern of co-infections. This suggests that while HPV-16-18 co-infections may be found at increased frequencies amongst US women, in South African adolescents, this specific HPV-16-18 relationship may not persist. Specifically, there was only a single case of HPV-16-18 co-infection (representing 8% of HPV-16 infections and 13% of HPV-18 infections). Additionally, HPV-18 infection alone (n=3) occurred at rates almost as high as in co-infections (n=5).

Our analysis found that not only were HPV-16 and HPV-18 co-infections weakly linked, but HPV-18 and HPV-68 infections (other HR-HPV genotypes found at increased frequency in adult African women with invasive cervical cancer) were mutually exclusive (Ogembo et al., 2015). HPV-68 was also rarely found as a single infection (only two of ten cases) and was not found among individuals of the disperse infections group. Additionally, individuals with an HPV infection by another HR-HPV alpha-7 species other than HPV-18 or HPV-68 were more likely to be found in the HPV-68 and alpha-7 group as compared to the Disperse Infections group (where they could also be infected with HPV-18).

South African women experience a disproportionate number of both HPV and HIV infections. In a meta-analysis that examined HPV infections in women with HIV, HIV-infected women were ten times more likely to be infected with multiple HPV genotypes than their HIV-uninfected counterparts (Clifford et al., 2006). HIV co-infection increases the risks for HPV persistence presence of multiple concurrent HPV infections and death from cervical cancer (Massad et al., 2001; Sun et al., 1997). In the present analysis, alpha-7 infections were the only pattern of infection significantly more prevalent among HIV-infected women. In the future, larger trials are needed to better understand other potential differences in HPV clustering as related to HIV-infection status and cytology results in cervical cancer screening.

This study is limited by its relatively small sample size. Although this is the first study to analyze HPV-clustering patterns in a particularly vulnerable population that should be the target of future vaccination programs in South Africa, larger studies are needed to strengthen the findings presented here. Understanding the patterns of HPV infection among high-risk groups is informative to HPV vaccination and screening strategies.

## Figures and Tables

**Table 1:** Demographic and behavioral characteristics of adolescent South African females infected with at least one HPV genotype.

Characteristics	Value
Average age (years)	19
% HIV-infected	64
**Average number of lifetime sexual partners**	**No. (%)**
1	11/64 (17.2)
2–5	47/64 (73.4)
>5	6/64 (9.4)
**Frequency of condom use**	
Always	28/64 (43.8)
Most of the time	17/64 (26.6)
Hardly ever	16/64 (25.0)
Never	3/64 (4.7)
**Form of contraception**	
None	2/64 (3.1)
Condom	55/64 (85.9)
Injection	40/64 (62.5)
Pill	3/64 (4.7)

**Table 2:** Latent class analysis of HPV clustering patterns in adolescent females in South Africa*.

HPV genotype(s)	Class 1: Uninfected (35%)	Class 2: Disperse infections (34%)	Class 3: HPV-68 and non-HPV-18 alpha-7 (18%)	Class 4: HPV 16, not HPV-18 (14%)
HPV-16	0.00	0.20	0.00	1.0
HPV-18	0.00	0.37	0.00	0.00
HPV-68	0.00	0.00	0.45	0.57
Other alpha-9 HPV (HPV-31, 33, 35, 52, 58 and/or 67)	0.00	0.51	0.00	0.22
Other alpha-7 HPV (HPV-39, 45, and/or 59)	0.00	0.43	0.60	0.24

* Values listed in Table II represent the conditional probably of infection with the given HPV genotype(s) given class membership
